# TSG-6 Activated MSC-derived Extracellular Vesicles Present Altered micro-RNA Contents and Ameliorate the Inflammatory Phenotype of Macrophages in Vitro

**DOI:** 10.1007/s10753-025-02398-y

**Published:** 2026-01-13

**Authors:** Iker Martinez-Zalbidea, Alyssa Rzasa, Varun Puvanesarajah, Wolfgang Hitzl, Karin Wuertz‑Kozak

**Affiliations:** 1 Department of Biomedical Engineering, Rochester Institute of Technology (RIT), Rochester, NY USA; 2 Department of Orthopedics and Rehabilitation, University of Rochester Medical Center, Rochester, NY USA; 3 Research and Innovation Management (RIM), Paracelsus Medical University, Salzburg, Austria; 4 Department of Ophthalmology and Optometry, Paracelsus Medical University, Salzburg, Austria; 5 Research Program Experimental Ophthalmology and Glaucoma Research, Paracelsus Medical University, Salzburg, Austria; 6 Schön Clinic Munich Harlaching, Spine Center, Academic Teaching Hospital and Spine Research Institute of the Paracelsus Medical University Salzburg (Austria), Munich, Germany

**Keywords:** Mesenchymal stem cells, Extracellular vesicles, Macrophage, polarization, Micro-RNA, Transcriptomic modulation

## Abstract

Mesenchymal stem cell-derived extracellular vesicles (MSC-EVs) have shown promising immunomodulatory properties; however, strategies to enhance their therapeutic potential remain limited. Here, we employed CRISPR activation of the gene TSG-6 in MSCs to evaluate the impact of elevated TSG-6 on EV cargo and immunomodulatory function in an in vitro macrophage model. CRISPR-mediated gene activation was confirmed by RT-qPCR, demonstrating more than an 1800 fold increase in TSG-6 mRNA compared to controls. EVs were isolated from TSG-6 overexpressing MSCs and thoroughly characterized by nanoparticle tracking analysis, transmission electron microscopy, and Western blot, confirming their typical size distribution, morphology, and surface markers. Small RNA sequencing of these EVs revealed 15 differentially expressed miRNAs relative to EVs from control MSCs. When THP-1–derived macrophages were stimulated with LPS and treated with TSG-6-overexpressing MSC-EVs (Standard dosage: 1000 particle/cell, *n* = 11; Alternative dosages: 500, 1000, or 2000 particles/cell, *n* = 6), a marked reduction in pro-inflammatory cytokine gene expression (IL-1β, CCL2, CXCL10, and TNF-α) and secreted protein levels (CCL2, TNF-α, CXCL1, and MIP-3α) was observed. Taken together, these findings demonstrate that CRISPR-based TSG-6 activation reprograms MSC-EV miRNA cargo (as well as their protein cargo, as previously shown), which can boost their anti-inflammatory effects. These findings underscore the promise of CRISPR-activation as a novel platform for boosting the bioactive properties of MSC-EVs and enhancing immunotherapeutic efficacy.

**Supplementary Information:**

The online version contains supplementary material available at 10.1007/s10753-025-02398-y.

## Introduction

Mesenchymal stem cells (MSC) are one of the more promising tools in regenerative medicine. Mounting evidence suggests that much of their observed therapeutic effect is due to the secretion of a wide range of trophic factors that can induce regenerative, immunomodulatory, anti-apoptotic, anti-catabolic, and anti-oxidative effects [1–4]. The therapeutic MSC secretome is composed of a wide range of bioactive molecules (including cytokines, chemokines, and growth factors) that can be released as free soluble factors, or encapsulated in lipid-bound vesicles known as Extracellular Vesicles (EVs) [1–6]. EVs are released by all cell types in both eukaryotic and prokaryotic organisms and can be extracted from different body fluids and cell culture conditioned media [7, 8]. EVs can be classified into different categories (such as exosomes, microvesicles, or apoptotic bodies) based on their biogenesis route, size, function, or composition, but the lack of specific markers and size overlap makes identifying each sub-fraction challenging [7, 9]. Therefore, in accordance with the recommendations from the Minimal Information for Studies of Extracellular Vesicles (MISEV) guidelines, we will collectively refer to them by the generic term “Extracellular Vesicles” or “EVs” [7, 10]. Released EVs can be taken up by other cells, thereby exerting important cell-to-cell communication roles. They serve as nanocarriers for bioactive molecules such as proteins, lipids, and nucleic acids (including DNA, mRNA, miRNA, and lncRNA) [7–9, 11]. The impact of EVs can differ significantly depending on the “donor” and “recipient” cells, their location within the organism, and the physiological context, with effects ranging from therapeutic to pathogenic [9, 12, 13]. MSC-EVs have demonstrated a wide range of therapeutic effects, including immune regulation, tissue repair promotion, and enhancement of cell survival in numerous different tissues, including cartilage, skin, bone, and intervertebral disc, among others [5, 8, 14–20]. A key mechanism by which MSC-EVs modulate the immune response is through their interaction with macrophages. Environmental signals, such as the secretion of pro-inflammatory cytokines, can recruit macrophages into sites of injury, where they play vital roles. Macrophage phenotypes are broadly classified into distinct polarization states: non-polarized M0 macrophages, which can differentiate into other states; classically activated M1 macrophages, which drive pro-inflammatory immune responses; and alternatively activated M2 macrophages, which promote anti-inflammatory effects and tissue repair [21–24]. The M1 macrophage phenotype is typically induced by classical activation signals such as lipopolysaccharide (LPS) or interferon-gamma (IFN-γ) [25–27]. These macrophages are characterized by the production of pro-inflammatory cytokines like tumor necrosis factor-alpha (TNF-α) and interleukin-6 (IL-6) [25, 28, 29]. In contrast, the M2 phenotype is driven by alternative activation signals such as interleukin-4 (IL-4) and interleukin-13 (IL-13) [22–26, 30, 31]. M2 macrophages are involved in tissue repair, fibrosis, and the resolution of inflammation, largely through the secretion of anti-inflammatory cytokines like IL-10 and transforming growth factor-beta (TGF-β) [28, 32, 33]. Current research suggests that macrophage polarization exists along a spectrum, with intermediate states and diverse functional outputs depending on the microenvironment and specific stimuli [31]. Macrophage polarization is not only essential for acute inflammatory responses but also plays a critical role in chronic diseases such as cancer, cardiovascular disease, and autoimmune disorders, where dysregulated macrophage activity can contribute to disease progression [28, 34, 35].

MSCs, particularly those derived from tissues such as bone marrow, adipose tissue, and umbilical cord, have been shown to modulate macrophage polarization in a variety of settings, including inflammation, injury, and chronic disease [36]. The immunosuppressive effects of MSCs are primarily mediated through the secretion of soluble factors, (including cytokines and growth factors), EVs, as well as through direct cell-to-cell interactions [37–41]. In the context of macrophage polarization, MSCs can skew macrophages towards an M2 phenotype, thereby promoting anti-inflammatory cytokine production, tissue remodeling, and resolution of inflammation [36, 42–45]. This polarization shift is particularly beneficial in conditions such as autoimmune diseases, chronic inflammation, and tissue injury, where an M2-like phenotype can promote healing and restore homeostasis [28, 35].

Recent studies have highlighted several signaling pathways and molecular mediators involved in MSC-induced macrophage polarization, including prostaglandins, transforming growth factor-beta (TGF-β), interleukin-10 (IL-10), and indoleamine 2,3-dioxygenase (IDO) [41, 46–52]. Additionally, MSC-derived EVs have emerged as important players in the communication between MSCs and macrophages, carrying bioactive cargo that can further modulate macrophage function [15, 36, 40, 45, 53]. The ability of MSC-EVs to regulate macrophage polarization presents a promising avenue for therapeutic interventions aimed at modulating immune responses in various diseases, from inflammatory conditions to cancer and tissue fibrosis [15]. In this context, the interaction between MSC-EVs and macrophages can be a target for testing new therapies, and macrophage models could make for an interesting bioassay to assess the potency of MSC-EVs [54].

THP-1-derived macrophages are a popular model for testing the effects of EVs in immunomodulation due to their similarity to primary human macrophages, their ease of differentiation, and their responsiveness to inflammatory stimuli [55–59]. THP-1 cells are a human monocytic leukemia cell line that can be differentiated into macrophage-like cells using phorbol 12-myristate 13-acetate (PMA), and demonstrate common macrophage characteristics [60, 61]. THP-1-derived macrophages are highly responsive to inflammatory stimuli such as LPS and cytokines (e.g., TNF-α, IL-1β), which allows for the evaluation of the immunomodulatory effects of MSC-EVs in a controlled, reproducible setting [55–59].

While MSC-EVs demonstrate clear therapeutic potential, their clinical translation is hindered by several factors, including limited therapeutic efficacy, variability between donors, and the heterogeneity of EVs [7, 12, 13, 62]. Several different approaches are currently being studied to improve MSC-EV-based therapies, including reducing EV heterogenicity, or boosting EV production. For example, hTERT immortalized MSC lines can be expanded long-term, which makes them well suited for as a standardized source of MSC-EVs, thus limiting the donor-variability observed when using multiple different sources of primary MSCs [63].

Additionally, MSC culture conditions can be altered using mechanical cues and substrate stiffness, changing cell seeding densities, using 3D scaffolds, or using other biophysical cues [3, 12, 64, 65]. In this context, substrate stiffness, topography, hydrostatic pressure, compression, and fluid flow can regulate MSC differentiation capacity, growth rate and EV secretion [64, 66–68]. Another approach is “priming” the cells using different types of biochemical cues. Bacterial LPS stimulation, or inflammatory cytokine treatments have been shown to increase the production of EVs and alter their immunomodulatory cargo [55, 56, 69–71].

In addition to MSC immortalization, a more cutting-edge approach is to reprogram MSCs for altering the EV cargo. For example, a study reported that overexpressing GATA-4 on MSCs altered the micro RNA (miR) cargo of MSC-EVs into a more cardioprotective profile [72]. Another study induced the overexpression of HIF-1α in MSCs and observed increased release of EVs and angiogenic effects [73].

An innovative approach is using CRISPR-based genomic engineering to alter EV cargo. Catalytically “dead” Cas9 (dCas9) variants of the CRISPR-Cas9 system can be coupled to transcription activators or repressors, and change the transcriptional expression of genes of interest [74–77]. One of the most robust systems to induce genetic overexpression is the CRISPR sgRNA-directed “synergistic activation mediator” (SAM), which combines a complex of dCas9 and MS2 fusion proteins [77].

We have previously used this CRISPR-based system to generate an MSC line overexpressing gene TSG-6 using this system [78]. We selected TSG-6 as the target for overexpression due to its fundamental role as a mediator in the anti-inflammatory effects of MSCs [79–81]. It functions as an autocrine factor that impacts a wide range of properties of MSCs by modulating transcription factors, cytokine expression, and other critical biological pathways and mechanisms [79–82]. Research has shown that TSG-6 is crucial to the therapeutic effects of MSC-derived EVs [82–85]. The TSG-6 overexpressing MSC line we generated presented significantly higher (> 800 fold) gene expression relative to controls, and protein levels were similarly higher in the cells and harvested MSC-EVs [78]. Furthermore, proteomic analysis demonstrated that the overexpression of TSG-6 resulted in altered peptide contents in EVs from TSG-6 “activated” cells (TSG-6 EVs) relative to controls [78]. In our previous work, we furthermore assessed the therapeutic effects of the reprogrammed TSG-6 EVs in an in vitro inflammatory model on human intervertebral disc (IVD) cells, observing significant downregulation of inflammatory markers IL-8 and COX-2 [78].

Given the central role of macrophages in various pathologies and the observed anti-inflammatory effects of TSG-6–activated MSC-EVs on IVD cells, we sought to further investigate their impact in a macrophage in vitro model. The relevance of this research is substantiated by numerous studies demonstrating that MSC-EVs exert therapeutic effects, at least in part, through TSG-6 signaling, with macrophages as a primary target cell [83, 84, 86–89]. Additionally, modifications of the EV miRNA content affect - and can enhance - the therapeutic potential of EVs [57, 62, 72]. Building on our previous work, we hence aim to determine whether CRISPR-based TSG-6 activation alters the miRNA cargo of MSC-EVs in a way that enhances their immunomodulatory effects. Specifically, we ask whether these reprogrammed EVs can shift macrophage polarization toward an anti-inflammatory phenotype, providing a potential therapeutic strategy for inflammatory conditions.

## Methods

### Human MSC Culture

The human immortalized MSC line “ASC52telo” (ATCC^®^ SCRC-4000™) was seeded at a density of 5,000 cells per cm^2^ in cell culture media (MEM Alpha, Cytiva, SH30024) supplemented with 10% fetal bovine serum (FBS, Cytiva, SH30396) and 1% antibiotic – antimycotic (anti-anti, Gibco, 15240062). The culture medium was replaced every 2–3 days, and cells were passaged when they reached approximately 80% confluence. For all experiments, MSCs were kept under 18 passages. In addition to the unmodified ASC52telo “wild-type” cells (WT), two CRISPR-modified ASC52telo cell lines were also used, “TSG-6 Activated” MSCs and “Non-Target control” (NTC) MSCs. These CRISPR-modified cells were generated by us as previously described [78]. Briefly, we transduced MSCs with the “SAM CRISPR Activator Lentivirus” (CRISPR Custom Lentiviral Vector, Sigma-Aldrich) system including single guide RNAs (sgRNA) targeting the promotor region of the gene of interest TNFAIP6 (TSG-6). We also generated a NTC cell line using non-targeting sgRNA as negative controls. Transduced cells were selected through antibody resistance and the efficiency of the genetic activation was determined by RT-qPCR and Western Blot.

## Collection of EVs

Before harvesting the EVs, MSCs lines (“TSG-6 activated”, “NTC” controls, or “WT” controls) were seeded and cultured following standard practices, as described above. Then medium was aspirated, and cells were washed twice with PBS (Cytiva, SH30028). Cells were then maintained for 48 h with serum-free basal media, after which the conditioned media (CM) was harvested from the MSC culture. The harvested CM was centrifuged first at 300 x g for 5 min and then subsequently at 1,500 x g for 15 min at 4 °C to remove cellular debris and large particles. The CM was then concentrated with 100,000 Nominal Molecular Weight Limit Amicon Ultra-15 centrifugation filters (Merck Millipore, UFC901024) by centrifugation at 2500 x g for 30 min at 4 °C. The resulting concentrates were then mixed with 0.5 volumes of Total Exosome Isolation Reagent (Invitrogen, 4478359) and vortexed until homogeneous. The mix was incubated overnight at 4 °C and was then centrifuged at 10,000 x g for 1 h at 4 °C. The final pellets were resuspended in 0.5 ml of PBS. EV particle concentration was quantified for each batch using nanoparticle tracking analysis (NTA), and only preparations from MSCs below passage 18 were included. All EV batches were isolated using identical protocols to ensure consistency across experiments.

## Characterization of EVs

EVs harvested from each MSC line (“TSG-6 Activated”, “NTC” and “WT”) were characterized for size-distribution, morphology, and molecular markers. Particle numbers and size-distribution were determined by nanoparticle tracking analysis (NTA) using a Nanosight 300 instrument following the manufacturer’s protocol. Briefly, MSC-EV suspensions were diluted 1:40 and analyzed with the instrument by taking 30-second recordings (*n* = 3) and analyzed with NanoSight NTA 3.4 software. EV particles were imaged by transmission electron microscopy (TEM) at the Electron Microscopy Shared Resource Laboratory at the University of Rochester Medical Center (URMC). Briefly, 3 µl of the EV suspension were placed in a 200-mesh carbon formvar coated copper grid (Electron Microscopy Science, Pennsylvania, USA) following glow discharge for 30 s at 30 mA and incubation for 30 s. Samples were blotted and grids were rinsed sequentially with molecular-grade water and a 0.75% solution of uranyl formate. Grids were air dried and imaged on a Hitachi 7650 transmission electron microscope with an NanoSprint 12 camera system (AMT).

The presence of common EV markers was characterized by Western Blot (WB). MSC-EVs were harvested as described above and the resulting pellets were lysed in 100 µl of RIPA Lysis and Extraction Buffer supplemented with 100× phosphatase and protease inhibitor cocktail, diluted to a final concentration of 1× in RIPA buffer (Thermo Fisher Scientific, 89901 and 78444).) and protein content was quantified by BCA assay (Thermo Fisher Scientific, 23225). EV protein samples (10 µg) were combined with 4X Laemmli buffer and 10% β-mercaptoethanol (Sigma Aldrich, 444203), heated on a heat block (95 °C, 5 min), and loaded onto a Mini-PROTEAN TGX 7.5% protein gel (Bio-Rad, 4568024) with a protein ladder (Thermo Fisher Scientific, 26617), in SDS-PAGE running buffer. Gel electrophoresis was run on Mini-PROTEAN Tetra Cells (Bio-Rad) and blots were transferred onto a 0.2 μm polyvinylidene difluoride (PVDF) Transfer-Blot Turbo Transfer Pack membrane (Bio-Rad, 1704156) in a Trans-Blot turbo transfer system (Bio-Rad, 1704150).

Transferred membranes were blocked in filtered 5% milk (Research Products International, M17200-500.0) with Tris-buffered saline-Tween (TBS-T) for 1 h. After washing in TBS-T, membranes were incubated with primary antibodies (TSG101 rabbit monoclonal, Abcam, EPR7130(B), ab125011; CD9 rabbit monoclonal, Abcam, EPR23105-125, ab263019; Calnexin rabbit monoclonal, Abcam, EPR3633(2), ab133615) diluted 1:1000 in 5% bovine serum albumin (Sigma-Aldrich) in TBS-T overnight at 4 °C. The following day, membranes were washed and incubated with secondary antibodies (Anti-rabbit IgG HRP-linked Antibody, Cell Signaling Technology, 7074 S) diluted 1:2000 in TBS-T for 1 h at room temperature. Protein bands were imaged using a chemiluminescence substrate (SuperSignal West Femto Maximum Sensitivity Substrate, Thermo Fisher Scientific, 34095) on an ODYSSEY XF Imaging System (LICOR).

## MiR Extraction, Sequencing and Bioinformatics Analysis

MSC-EV RNA was extracted with the Cell Culture Media Exosome Purification and RNA Isolation Mini Kit (Norgen Biotek, 60700) following the manufacturer’s instructions. RNA concentration was determined using the Nanophotometer N50 instrument (Implen). Prior to sequencing, RNA concentration and quality were assessed using the NanopDrop 1000 spectrophotometer and Agilent Bioanalyzer 2100. 500ng of Total RNA was utilized as input for sequencing library construction with the Illumina TruSeq Small RNA Library Prep kit (Illumina), following the manufacturer’s manual. Initially, 3’ and 5’ adaptors were diluted 2 fold for ligation, following 3’ adaptor ligation, RT primer incubation, and 5’ adaptor ligation. cDNA was synthesized, and library amplification was performed with 12 cycles of PCR. Library quantity and quality were determined with a Qubit fluorometer (ThermoFisher) and Tapestation 4200/Bioanalyzer/Fragment Analyzer (Agilent). Sample libraries were proportionally pooled and isolated from the PCR reaction mix using the Qiagen MinElute PCR Reaction Cleanup kit. Library fragments between 125 and 160 bp were size-selected using a 3% cassette on a PippinHT (Sage Science). The size-selected library pool was then sequenced on an Illumina NextSeq 2000, generating single-end reads of 50nt.

The raw reads generated from the Illumina basecalls were demultiplexed using bcl-convert version 4.1.7 [90]. miRge2.0 (2.0.3) was used to align reads with bowtie (1.2.1.1) and identify miRs with the following project-specific parameters: miRge2.0 annotate -s fileList -d miRBase -sp human -ad TGGAATTCTCGGGTGCCAAGG [91]. The differential expression analysis was performed using DESeq2-1.34.0 with a P-value threshold of 0.05 within R version 4.0.2 (https://www.R-project.org/) [92]. PCA plot was generated within R using pcaExplorer c2.14.2 to measure sample expression variance [93]. Heatmaps were generated using the pheatmap 1.0.12 package was given the rLog transformed expression values [94]. Volcano plots and dot plots were created using ggplot2 [95].

The differential expression of each miR between EV group pairs “TSG-6 vs NTC”, “TSG-6 vs WT” and “NTC vs WT” was compared (*n* = 3). Only miRNAs with statistical p-values < 0.05 were considered for comparison. miRNAs with Log2 Fold Changes ≤ 1 were considered as being significantly downregulated, and miRNAs with values ≥ 1 were considered as significantly up-regulated. These miRNAs with altered expression levels were considered for further bioinformatic target and pathway prediction. To predict miRNA target genes and pathways, the bioinformatic miRabel software was used (http://bioinfo.univ-rouen.fr/mirabel/index.php). The miRNAs with altered expression profiles were ranked based on the miRabel tool results. This tool aggregates results from several miRNA databases (miRanda, PITA, SVmicrO, and TargetScan), as well as experimentally validated interactions (annotated with miRTarBase v.6.0 and miRecords), and 5’UTR and CDS predictions (using the mirWalk database) [96]. The top 100 ranked targets and pathways were screened for relevance in macrophage inflammatory processes. Additionally, the possible interactions between TSG-6 and the list of miR with altered expression levels were investigated with the miRabel tool, searching “by gene” TNFAIP6 (TSG-6).

To further investigate the mechanistic relevance of the six microRNAs significantly upregulated in EVs derived from TSG-6-overexpressing MSCs (hsa-miR-324-5p, hsa-miR-3130-3p, hsa-miR-2467-5p, hsa-miR-28-5p, hsa-miR-181a-5p, and hsa-miR-181b-5p; log2FC > 1, *p* < 0.05), we performed integrated target prediction using miRNet 2.0. Target genes were predicted using the miRTarBase v9.0 database. Protein-protein interaction networks were explored using the STRING interactome with experimentally validated data. To visualize the regulatory relationships among the six miRNAs, gene TSG-6 (TNFAIP6), and their shared target genes, we generated a miRNA–target–gene interaction network in miRNet. A subnetwork centered on TSG-6 and its protein interaction partners was extracted to highlight putative mechanistic links to miR network, respectively. In addition, to investigate the biological significance of the overexpressed miRNAs, pathway enrichment analyses were conducted using miRNet 2.0. Specifically, Kyoto Encyclopedia of Genes and Genomes (KEGG) pathway enrichment and Gene Ontology Biological Process (GO: BP) enrichment analyses were performed to identify significantly overrepresented pathways. Statistical significance was determined using an adjusted p-value threshold (False Discovery Rate, FDR) of < 0.05.

## THP-1 Cell Culture and Macrophage Differentiation

Human THP-1 cells were (ATCC^®^ TIB-202™) were seeded in suspension at a density of 200,000 cell/ml in basal media (RPMI, Cytiva, SH30255) supplemented with 10% fetal bovine serum (FBS, Cytiva, SH30396), 1mM Sodium Pyrubate (Gibco, 11360-070) and 1% antibiotic – antimycotic (anti-anti, Gibco, 15240062). The culture medium was replaced every 2–3 days, and cells were passaged before they reached a density of 1 million cell/ml. Cells were maintained in standard suspension culture conditions in a humidified incubator at 37 °C, 5% CO2.

### Macrophage LPS Stimulation Model

400,000 THP-1 cells were seeded per well of a 12-well plate in RPMI cell culture media (supplemented with 10% FBS + 1% anti-anti + 1 mM Sodium Pyruvate, Gibco, 11360-070) treated with 100 ng/mL PMA (Cayman, 10008014) for 48 h. After the initial 48 h PMA treatment, THP-1 derived macrophages were stimulated with 500 ng/ml LPS (Sigma-Aldrich, L6529) in serum-free RPMI (+ 1% anti-anti) culture media. 2 h after LPS pre-stimulation, cells were co-treated with the previously isolated EV suspension for 24 h. 24 h after treatment, cell culture media supernatants and cell RNA were collected. Each experimental condition received a specific dosage of EV suspension particles from either “NTC”-EVs or “TSG-6”-EVs. In the main experimental setup, we used 1000 particles/cell (*n* = 11). In addition, a dose-dependency experiment with 3 different EV dosages (500, 1000 or 2000 particle/cell, *n* = 6) was conducted. Negative controls were fully untreated differentiated THP-1 cells. Additional controls included differentiated THP1 cells that received only LPS stimulation, or only EV treatment. Table 1 outlines the experimental conditions.

**Table 1:** General experimental setup for EV treatment and LPS pro-inflammatory co-treatment of macrophages. Basal medium for all experimental conditions was serum free basal cell culture medium (RPMI with 1% antibiotic – antimycotic + 1 mM sodium Pyruvate)

	Untreated control	LPS control	EV treatment	LPS + EV co-treatment
*Start of LPS stimulation*	Replace media	500 ng/ml LPS	Replace media	500 ng/ml LPS
*2 h post-stimulation*	Replace media	500 ng/ml LPS	EV treatment	EV treatment (500 ng/ml LPS + EV)
*24 h post-EV treatment*	Culture media supernatant and RNA collection			

## EV Uptake by Macrophages

To confirm the internalization of EVs into macrophages, we performed an uptake assay using MSC-EVs labelled with the PKH26 red fluorescent membrane kit (Sigma–Aldrich, USA, 4103849473). EV suspensions were harvested and dosages determined as previously described. For EV labeling, isolated EVs were first pelleted by ultracentrifugation (100,000 × g, 1 h, 4 °C) in an Optima MAX-XP ultracentrifuge (TLS-55 rotor, Beckman Coulter, USA, 346936) using 1 mL Open-Top Thickwall polycarbonate tubes (Beckman Coulter, USA, 343778). Afterwards, pellets were resuspended in Diluent C (Sigma–Aldrich, USA, 4103849473) and stained with PKH26 red fluorescent dye (4 µL in 2 mL Diluent C) for 10 min at room temperature, in the dark. The reaction was quenched in cell culture media (MEM Alpha, Cytiva, SH30024) supplemented with 10% fetal bovine serum (FBS, Cytiva, SH30396), and excess dye was removed via ultracentrifugation (100,000 × g, 1 h, 4 °C). The labeled EVs were resuspended in PBS and then pelleted by ultracentrifugation (100,000 × g, 1 h, 4 °C). THP-1 monocytes were plated in a 24-well plate at 200,000 cells/well and differentiated into M0 macrophages using 100 ng/mL PMA, for 48 h. After differentiation, M0 macrophages were polarized into an inflammatory (M1) phenotype using 500 ng/mL LPS, for 2 h, followed by treatment with PKH26-labeled EVs, 5,000 particles/cell) for 24 h. Cells were then fixed with 4% paraformaldehyde (PFA) (Thermo Scientific, USA, J19943-K2), permeabilized with 0.1% Triton X-100 (Alfa Aesar, USA, J63521), and blocked with 4% bovine serum albumin (BSA) (Thermo Scientific Chemicals, USA, AAJ6410009). Actin cytoskeletons were stained with Alexa Fluor 488 phalloidin: (Invitrogen, USA, A123790) (1:400 in BSA), and nuclei were counterstained with Hoechst 33342 (Thermo Scientific, USA, H3570) (1:3,000 in PBS). Samples were imaged using fluorescence microscopy (Olympus IX81, USA) and processed in FIJI (Image J) software.

## RNA Extraction and Gene Expression Analysis (RT-qPCR)

Cell lysis and mRNA extraction for gene expression analysis were performed with the RNeasy Mini Kit (Qiagen, 74104) following the manufacturer’s recommendations. The concentration and quality of mRNA were quantified with a Nanophotometer N50 instrument (Implen). Reverse transcription of RNA was achieved with the High-Capacity cDNA Reverse Transcription Kit with RNase Inhibitor (Thermo Fisher Scientific, 4374967) using 1000 ng of RNA. Gene expression was assessed by qPCR using the TaqMan™ Fast Advanced Master Mix (Thermo Fisher Scientific, 4444963) and Taqman™ Gene Expression Assay primers (Hs00427620_m1 TBP, Hs00200180_m1 TNFAIP6, Hs01555410_m1 IL-1β, Hs00174131_m1 IL-6, Hs00961622_m1 IL-10, Hs00174128_m1 TNF-α, Hs00234140_m1 CCL2, Hs00171042_m1 CXCL10, Thermo Fisher Scientific, 4331182) using the QuantStudio 3 instrument and software (Thermo Fisher Scientific). The results were calculated as 2^−∆∆Ct^ values relative to housekeeping gene TBP and control conditions.

### Cytokine Array

Macrophage cytokine secretion was analyzed with a Human Cytokine Antibody Array C5 (RayBiotech Life, AAH-CYT-5). Macrophage culture supernatants (From “Untreated”, “LPS control”, “NTC-EV co-treatment” and “TSG-6 EV co-treatment” conditions, *n* = 3) were collected after treatment and centrifuged at 1000 x g for 15 min at 4 °C in order to remove any debris (EV dosages: 1000 particle/cell). The resulting supernatants were used undiluted following the manufacturer’s user manual. Membrane chemiluminescence was imaged with the Odyssey XF imaging system (LI-COR Biosciences) and analyzed with the Empiria Studio Software (LI-COR Biosciences). The median fluorescent signal was normalized to the plate background and array’s positive control. Fold changes of fluorescence were calculated relative to the NTC treatment conditions.

### Statistical Methods

Independent Student t-tests and one sample t-tests and corresponding bootstrap-t tests based on 3000 Monte Carlo simulations were used to test means. Whisker plots with standard errors were used to illustrate results. All reported tests are 2-sided and p-values < 0.05 were considered as statistically significant. While multiple groups were included (e.g., IL-1β, EV treatment, co-treatment), primary comparisons focused on specific pairwise contrasts (e.g., LPS alone vs. LPS + EV). All statistical analyses in this report were performed by use of STATISTICA 13 (Hill, T. & Lewicki, P. Statistics: Methods and Applications. StatSoft, Tulsa, OK), MATHEMATICA (Wolfram Research, Inc., Mathematica, Version 13, Champaign, IL, 2022) and PASW 29 (IBM SPSS Statistics for Windows, Version 29.0., Armonk, NY).

## Results

### CRISPR-activated MSCs Retain TSG-6 Gene Overexpression in Late Passages

**Fig. 1:**
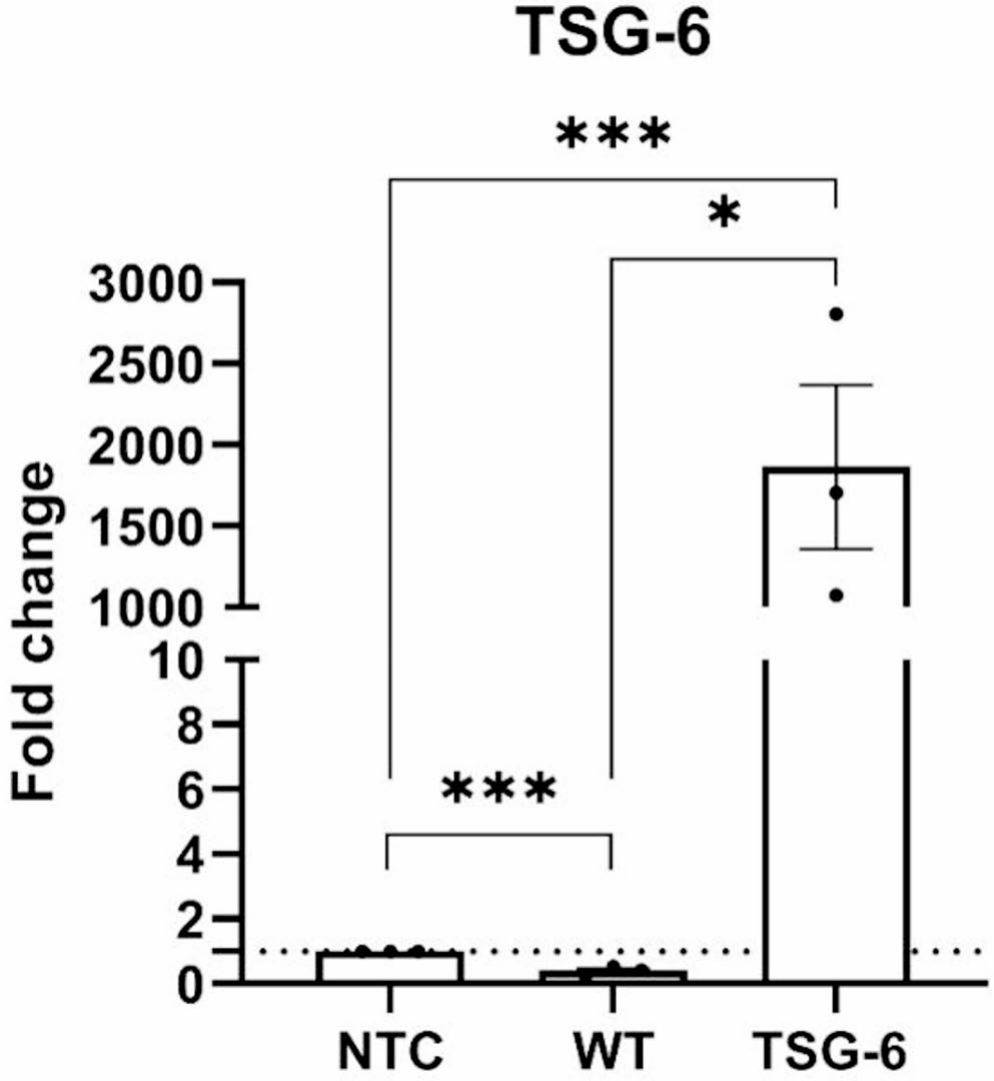
Gene expression of TSG-6 in NTC, WT and TSG-6 “activated” MSCs (*N* = 3). Mean ± SEM, *p < 0.05, **p < 0.01, ***p < 0.001

We previously reported the successful generation of an ASC52telo MSC-line overexpressing the gene TSG-6 [78]. For the current study, we validated the long-term stability of the achieved TSG-6 overexpression in late passage MSCs (passage 18), relative to control cells (*n* = 3). Even at late passage, the CRISPR-activated cells presented a significantly higher expression of TSG-6 (relative to NTC-MSCs (1861 ± 506.5 fold change, *p* = 0.001) and relative to WT-MSCs (1862 ± 506.5 fold change, *p* = 0.021) (Fig. 1). A small but statistically significant difference in TSG6 expression was observed between NTC and WT (0.60 ± 0.08 fold change, *p* = 0.01). This data confirms that genetic overexpression is retained long-term in this cell line.

### CRISPR-activated EVs Present Common EV Characteristics

**Fig. 2:**
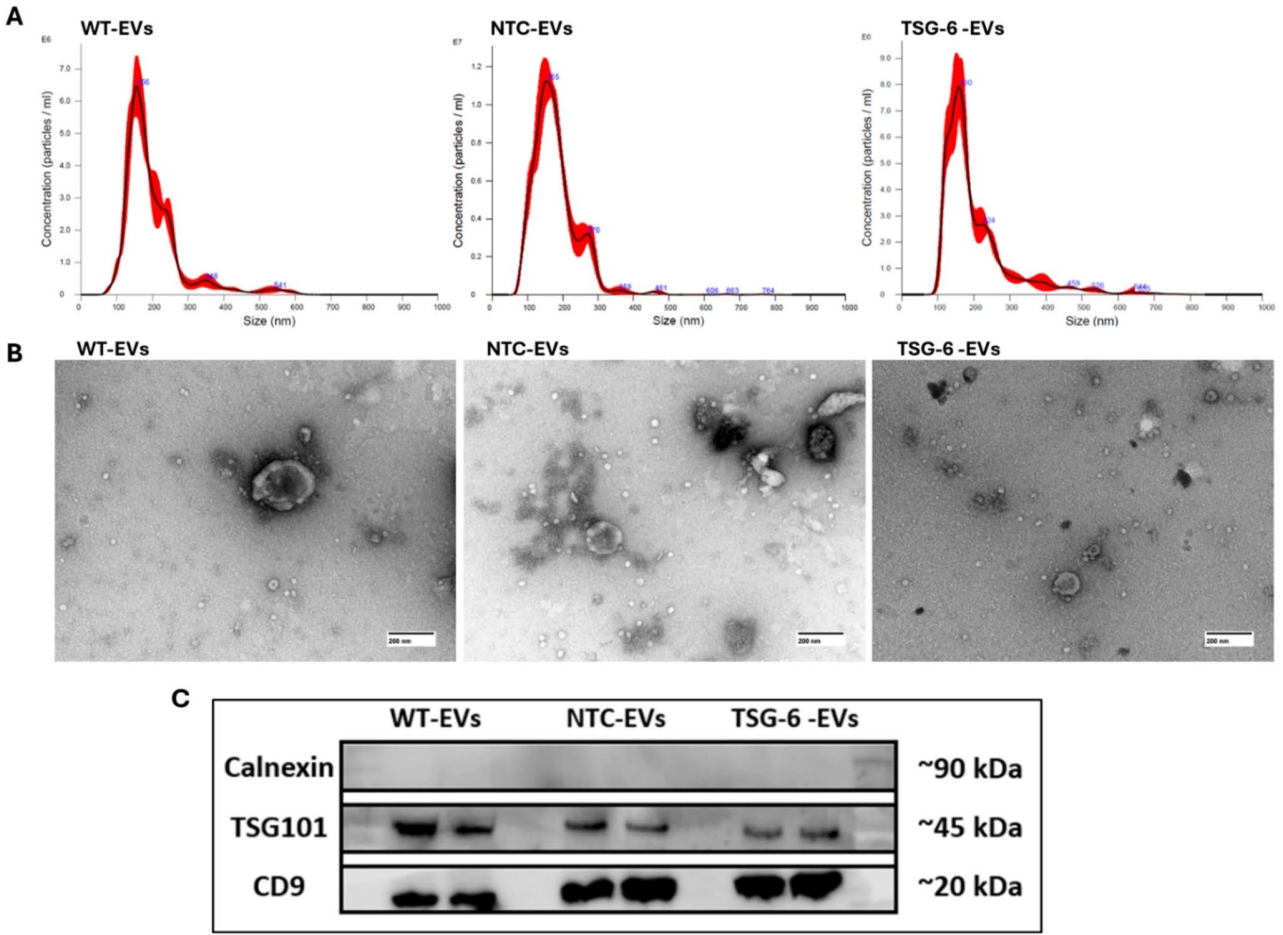
Representation of particle size distribution, morphology and protein markers from EV samples of CRSIPR-activated MSCs and controls. (**A**) Particle size-distribution analysis by NTA (WT-EVs: 199.5 ± 3.8 nm; NTC-EVs: 177.7 ± 1.9 nm; and TSG-6 -EVs: 204.6 ± 3.0 nm) (*n* = 3). (**B**) Representative TEM images of “EV-like” structures (*N* = 1. Scale bar = 200 nm). (**C**) Protein expression of Calnexin, TSG101 and CD9 in MSC-EVs (WT-EV, NTC-EV and TSG-6 “Activated” EVs), with technical duplicates (*n* = 2)

To further characterize MSC-EVs, we assessed EV size distribution, morphology, and the presence of molecular markers for EVs harvested from WT, NTC, and TSG-6 “activated” MSCs. NTA measurements showed similar size-distribution profiles between each EV source, as shown in Fig. 2A, and particle counts in the range of 1 × 10^9^ – 1 × 10^10^ particles/ml. TEM imaging of EVs revealed cup-like particles ≤ 200 nm in all samples (Fig. 2B). Additionally, we assessed the presence of molecular markers through WB and detected the presence of tetraspanin CD9 and cytosolic TSG101, proteins found enriched in EVs, and lacked the presence of Calnexin, an intracellular protein that is described as depleted in small EV samples (Fig. 2C). These results are comparable between TSG-6 “activated”, WT and NTC MSC-EVs, and consistent with what we reported previously and with what is shown in the literature [78]. Therefore, we can confirm that our CRISPR-activated MSCs produce EVs with similar characteristics to control cells.

### Macrophages Take Up Labelled MSC-EVs

**Fig. 3:**
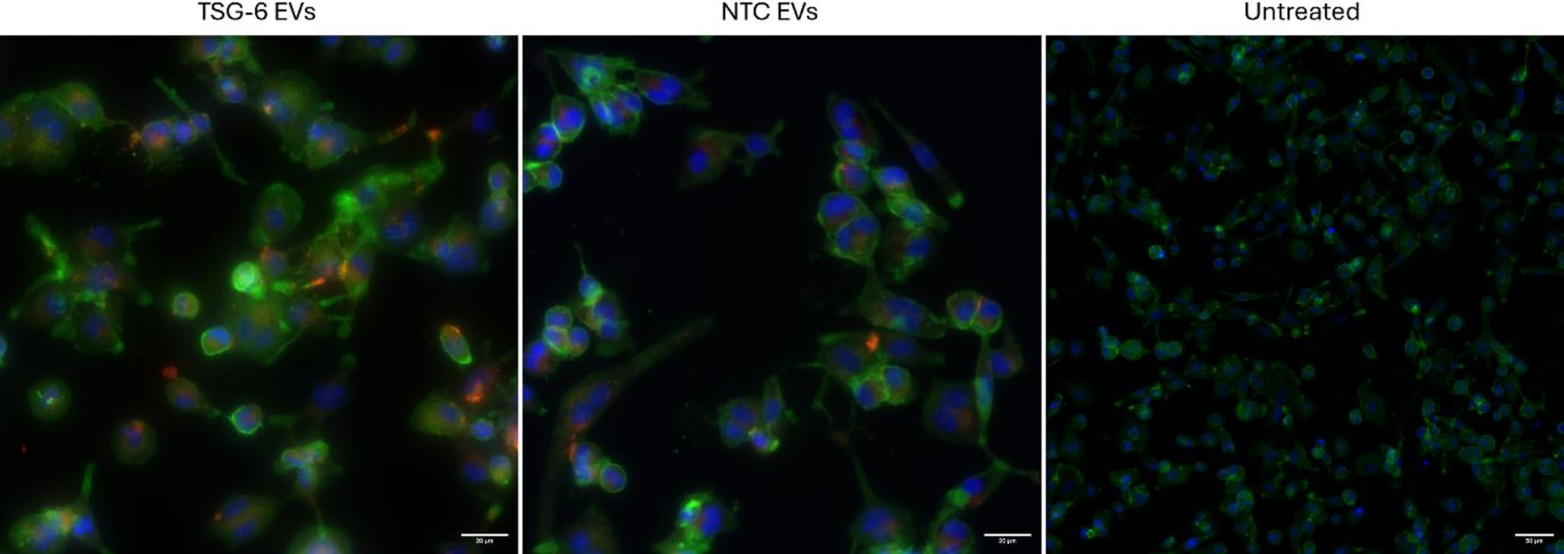
Representative immunofluorescence images showing the distribution of PKH26-labeled MSC-derived EVs after 24-hour co-treatment with EVs at a concentration of 5,000 particles per cell. The left and center panels show EV-treated samples (scale bar: 20 μm), while the right panel shows the untreated control (scale bar: 50 μm). Green: Alexa Fluor 488–phalloidin; Red: PKH26 fluorescent dye; Blue: Hoechst 33,342 nuclear stain

To observe the uptake of EVs into macrophages, we labelled MSC-EVs (TSG-6-activated and NTC controls) with red fluorescence PKH-26 and treated LPS pre-stimulated macrophages with the labelled EVs. After 24 h we imaged the macrophages by fluorescence. We detected the presence of red PKH26 signal in macrophages treated with TSG-6 activated EVs and NTC EVs (Fig. 3).

### CRISPR-activation Induces Transcriptomic Changes on MSC-EV Contents

**Fig. 4:**
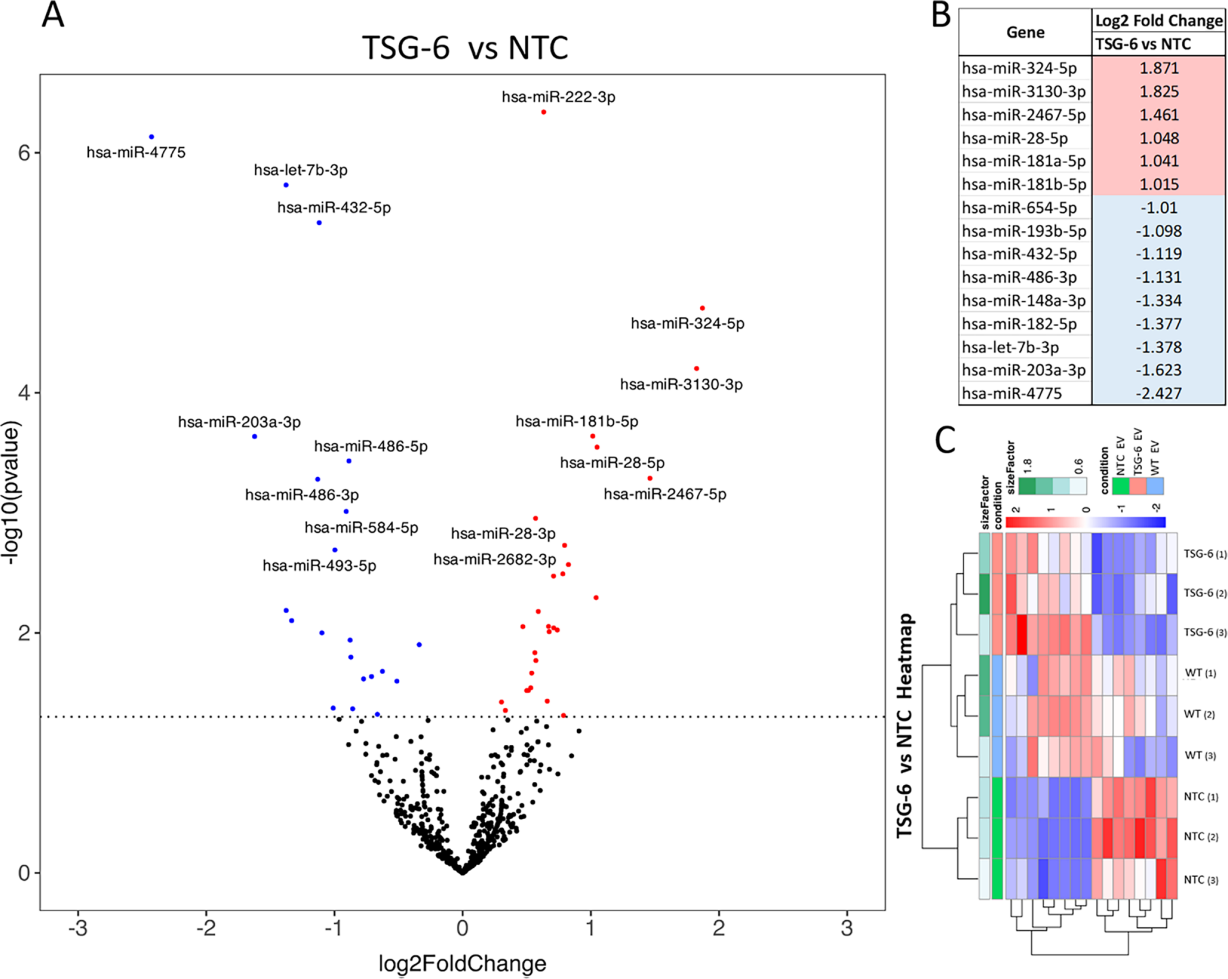
Identification of miR with altered expression levels in MSC-EVs, comparing “TSG-6 vs NTC”. (**A**) Volcano plot of MSC-EV miR contents. (**B**) List of significantly up-regulated and downregulated miRs for “TSG-6 vs NTC” comparison. (**C**) Heat map of sample size factors including all EV samples

miRNAs (miRs) with altered expression levels between EV groups were screened with the miRabel tool, ranking the results with the prediction software miRanda, PITA, SVmicrO, and TargetSacn. In the comparative analysis of EV-miR for the “TSG-6 vs NTC” comparisons, six miRs were significantly upregulated, and 9 miRs significantly downregulated. Figure 4 presents the differential expression of miRs in Volcano plot. Supplementary Fig. 1 includes additional Volcano plot comparisons and the full list of miRs can be found in Supplementary Spreadsheet 1.

We focused the search of impacted pathways on the miRs overexpressed for the “TSG-6 vs NTC” comparisons, and gene TSG-6. More specifically, the prediction of miR targets of gene TNFAIP6 (TSG-6) resulted in 308 entries, of these, “hsa-miR-181a-5p” was identified as being upregulated for the “TSG-6 vs NTC” comparisons. Predictions of “hsa-miR-181a-5p” targets in the human genome resulted in 9,675 possible target genes and 330 targeted pathways. We studied the top 100 ranked pathways and studied only relevant macrophage pathways (such as inflammatory pathways).

**Fig. 5:**
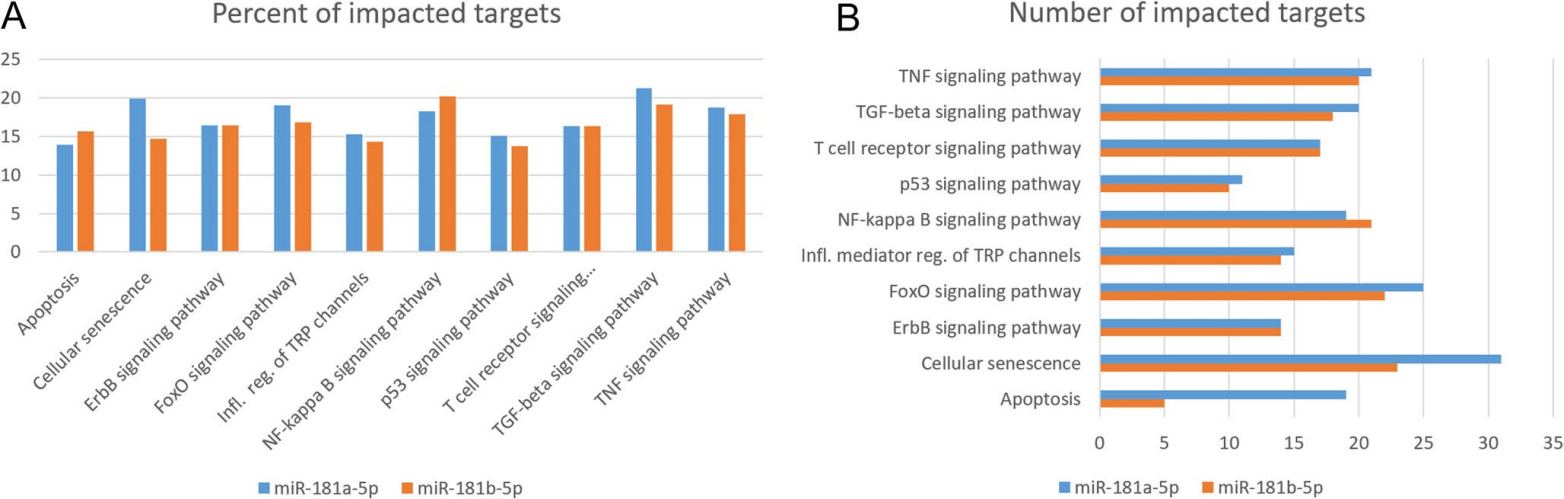
miR-181a-5p and miR-181b-5p target and pathway prediction. (**A**) Pathways targeted based on miR sequence in the number of targets per pathway. (**B**) Pathways targeted based on miR-181 sequence in % of genes targeted per pathway

The resulting macrophage-relevant pathway prediction showed potential regulation between hsa-miR-181a-5p and several inflammatory pathways, such as TGF-β, TNF-α, and NF-κB. Among the most relevant pathways, hsa-miR-181a-5p targets 21.28% of the genes of the TGF-beta signaling pathway, 19.87% of the cellular senescence pathway, 18.75% of the TNF-α signaling pathway, 18.27% of the NF-kappa B signaling pathway, 16.35% of the T cell receptor signaling pathway, and 15.31% of the inflammatory mediator regulation of TRP channels. Another up-regulated miR, hsa-miR-181b-5p, presents a similar pathway impact. The pathway impacts of these miRs are depicted in Fig. 5.

**Fig. 6:**
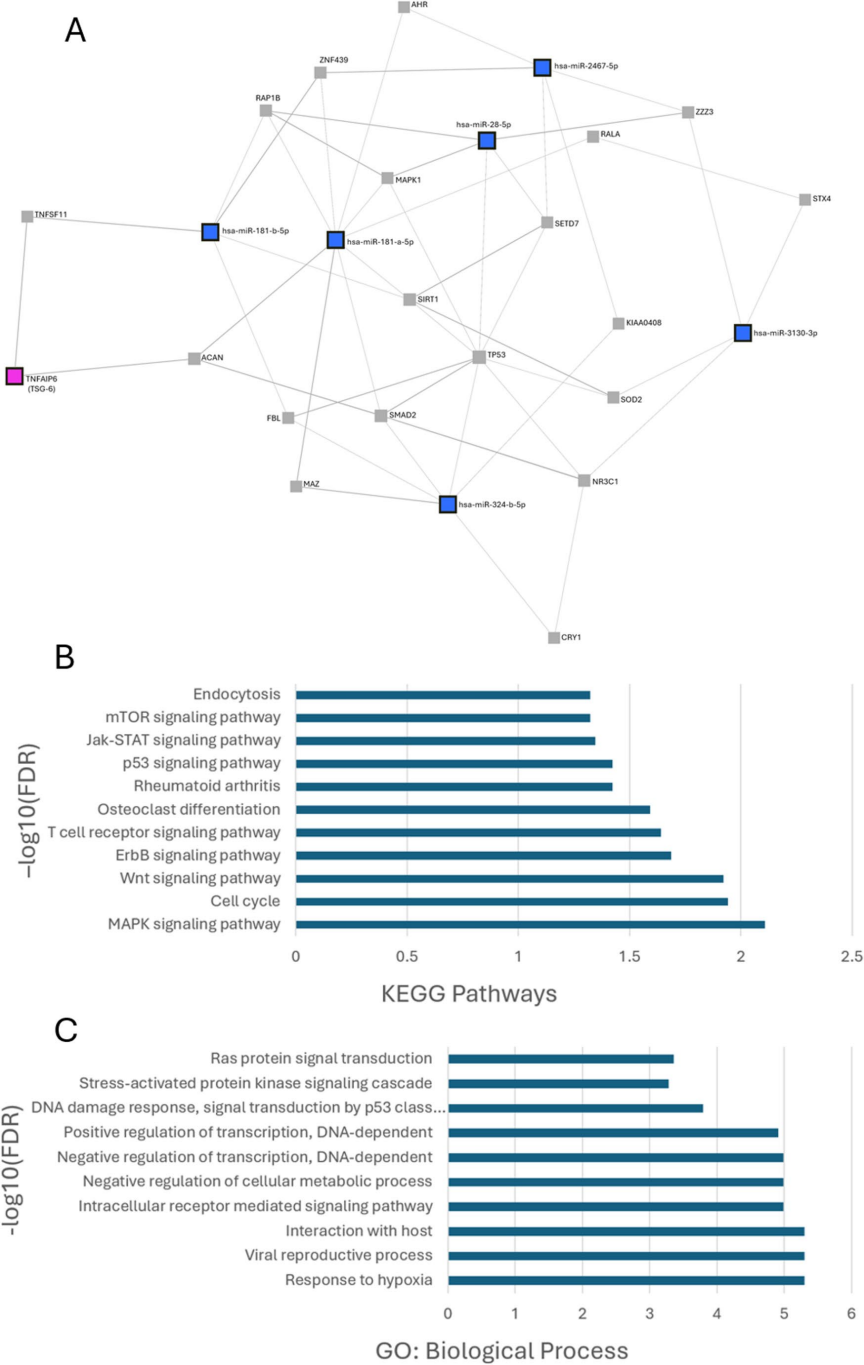
(**A**) miRNA–Target–Protein–Protein Interaction (PPI) Network Generated Using miRNet 2.0. The network incorporates the six key microRNAs enriched in EVs from TSG-6-activated MSCs (blue squares), their predicted or validated target genes (gray nodes), and TSG-6 (TNFAIP6, magenta square). Known PPIs from STRING are included. (**B**) KEGG enriched pathways. (**C**) GO enriched biological processes

To explore the biological relevance of microRNAs enriched in EVs from TSG-6-activated MSCs, we used miRNet 2.0 to construct a miRNA–target–protein interaction network. The six most upregulated miRNAs in TSG-6-EVs - including miR-181a-5p and miR-181b-5p - were entered into the network alongside TSG-6 (TNFAIP6). The resulting analysis revealed direct protein-protein interactions between TSG-6 and two targets, aggrecan (ACAN) and RANKL (TNFSF11), both of which are also targeted by miR-181 family members (Fig. 6A).

KEGG pathway enrichment analysis of the six overexpressed miRNAs revealed significant activation of immune and signaling pathways (Fig. 6B). The MAPK signaling pathway (FDR = 0.00781; –log10[FDR] = 2.11) was among the most enriched, with 34 observed hits compared to 19.9 expected, indicating robust pathway engagement. Other notably enriched pathways included cell cycle (FDR = 0.0114), Wnt signaling (FDR = 0.012), and T cell receptor signaling (FDR = 0.0229), reflecting altered proliferative and immune signaling dynamics. Pathways associated with macrophage function, such as osteoclast differentiation, Jak-STAT, mTOR, and ErbB signaling, were also significantly enriched (FDR < 0.05), suggesting involvement in cytokine-mediated responses and metabolic regulation. Additionally, enrichment of the p53 signaling pathway and endocytosis points to activation of stress responses and phagocytic activity.

GO analysis further supported these findings, highlighting enrichment of processes central to macrophage-mediated immunity (Fig. 6C). Highly significant terms included response to hypoxia and viral reproductive process (both FDR = 5.02 × 10⁻⁶), consistent with macrophage roles in infection and tumor microenvironments. Other enriched terms such as interaction with host, intracellular receptor-mediated signaling, and regulation of transcription (positive and negative) reflect complex regulation of immune activation. Processes linked to macrophage polarization and stress responses, such as negative regulation of cellular metabolism, DNA damage response, and the stress-activated protein kinase cascade, were also significantly enriched (FDR < 0.001).

These interactions place miR-181a-5p and miR-181b-5p as central hubs in the network, connecting to additional regulators of inflammation and tissue remodeling, including TP53, SMAD2, SIRT1, and MAPK1. The network supports the hypothesis that TSG-6 activation modulates EV miRNA cargo toward functional targeting of both immune pathways and extracellular matrix regulation.

### TSG-6 Activated EVs Reverse LPS Inflammatory Stimulation of Macrophages

**Fig. 7:**
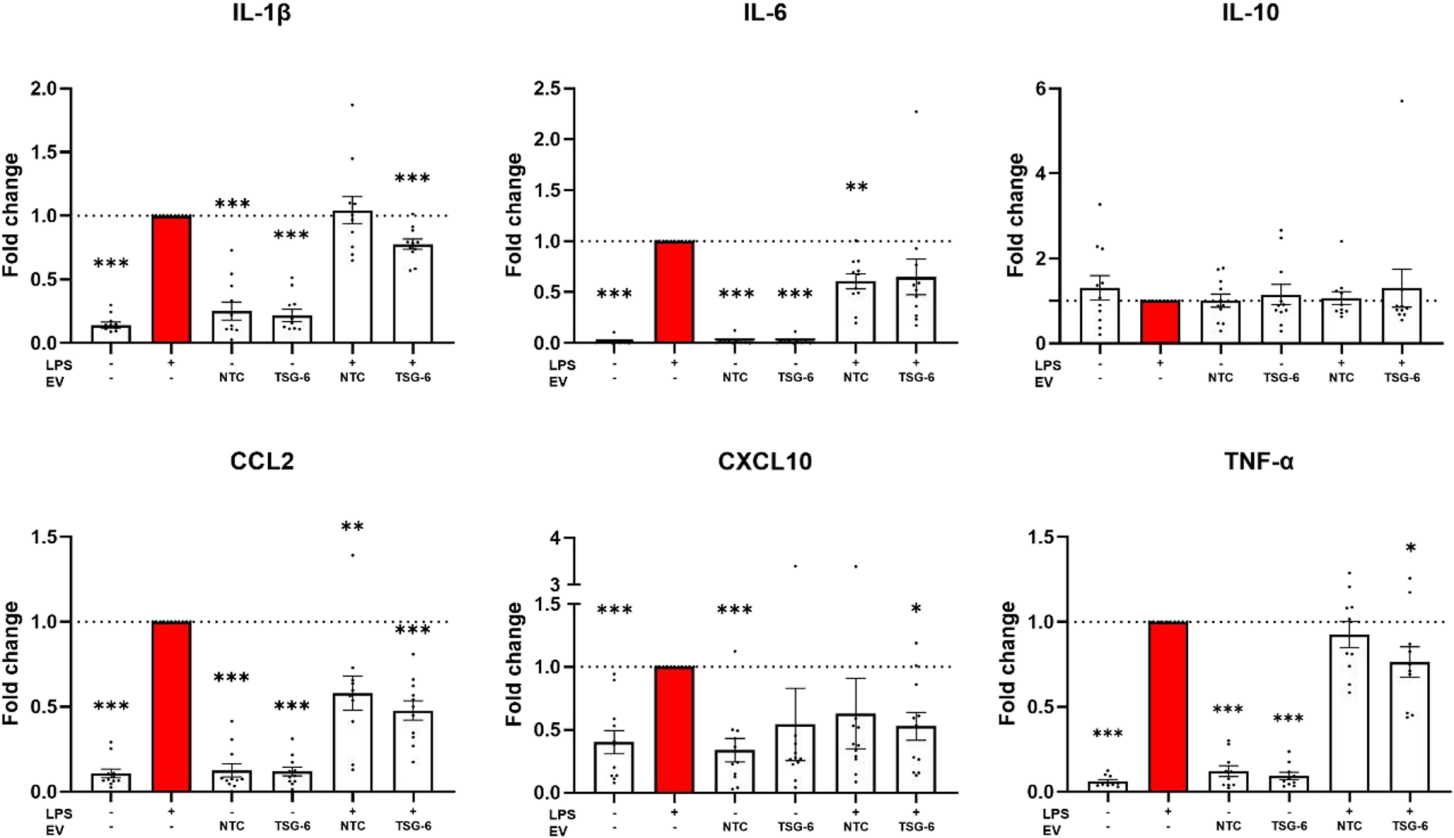
RT-qPCR data showing the effect of LPS and MSC-EV co-treatments on THP-1 derived macrophages (*N* = 11, *n* = 10 for TNF-α). Mean ± SEM, *p < 0.05, **p < 0.01, ***p < 0.001 relative to LPS treatment

As recent research indicates that both MSC-EVs and TSG-6 can modulate macrophage polarization, we sought to determine the immune-modulatory impact of EVs from TSG-6-MSCs and NTC-MSCs on LPS stimulated macrophages. LPS stimulation significantly up-regulated the expression of pro-inflammatory genes IL-1β, IL-6, CCL2, CXCL10 and TNF-α (Fig. 7) (*n* = 11, *n* = 10 for TNF-α). LPS co-treatment with TSG-6 “activated” EVs achieved a significant downregulation of genes IL-1β, CCL2, CXCL10, and TNF-α. While we observed a stronger downregulatory effect from TSG-6 EVs than NTC-EVs for several genes (IL-1β: *p* = 0.092, TNF-α: *p* = 0.188) these differences did not reach significance.

**Fig. 8:**
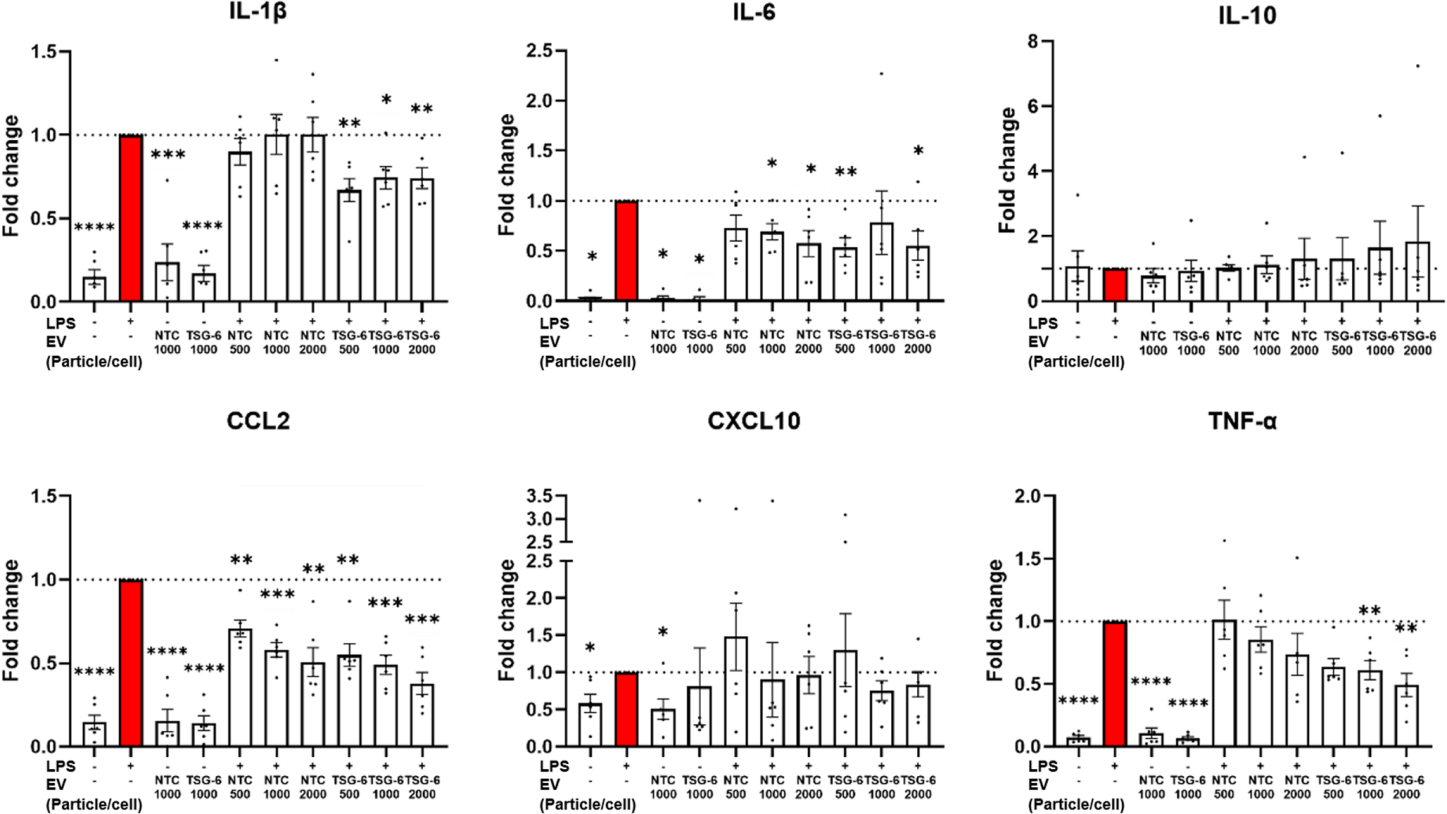
RT-qPCR data showing the effect of LPS and different MSC-EV co-treatments on THP-1 derived macrophages (EV dosages: 500 particle/cell, 1000 particle/cell, or 2000 particles/cell; *n* = 6). Mean ± SEM, *p < 0.05, **p < 0.01, ***p < 0.001 relative to LPS treatment

We furthermore ran a dose-dependency experiment, allowing us to compare the effect of EV dosage (500 particle/cell, 1000 particle/cell, and 2000 particles/cell; *n* = 6) as well as further asses potential difference between TSG-6 or NTC EVs at different dosage. Similar to data shown in Fig. 5, TSG-6 activated EVs presented a trend of superior downregulation effect relative to NTC-EVs (Fig. 8). Although no statistically significant dose-dependency effects were achieved, higher EV dosages seemed to have a more pronounced effect on the expression of CCL2 and TNF-α.

**Fig. 9:**
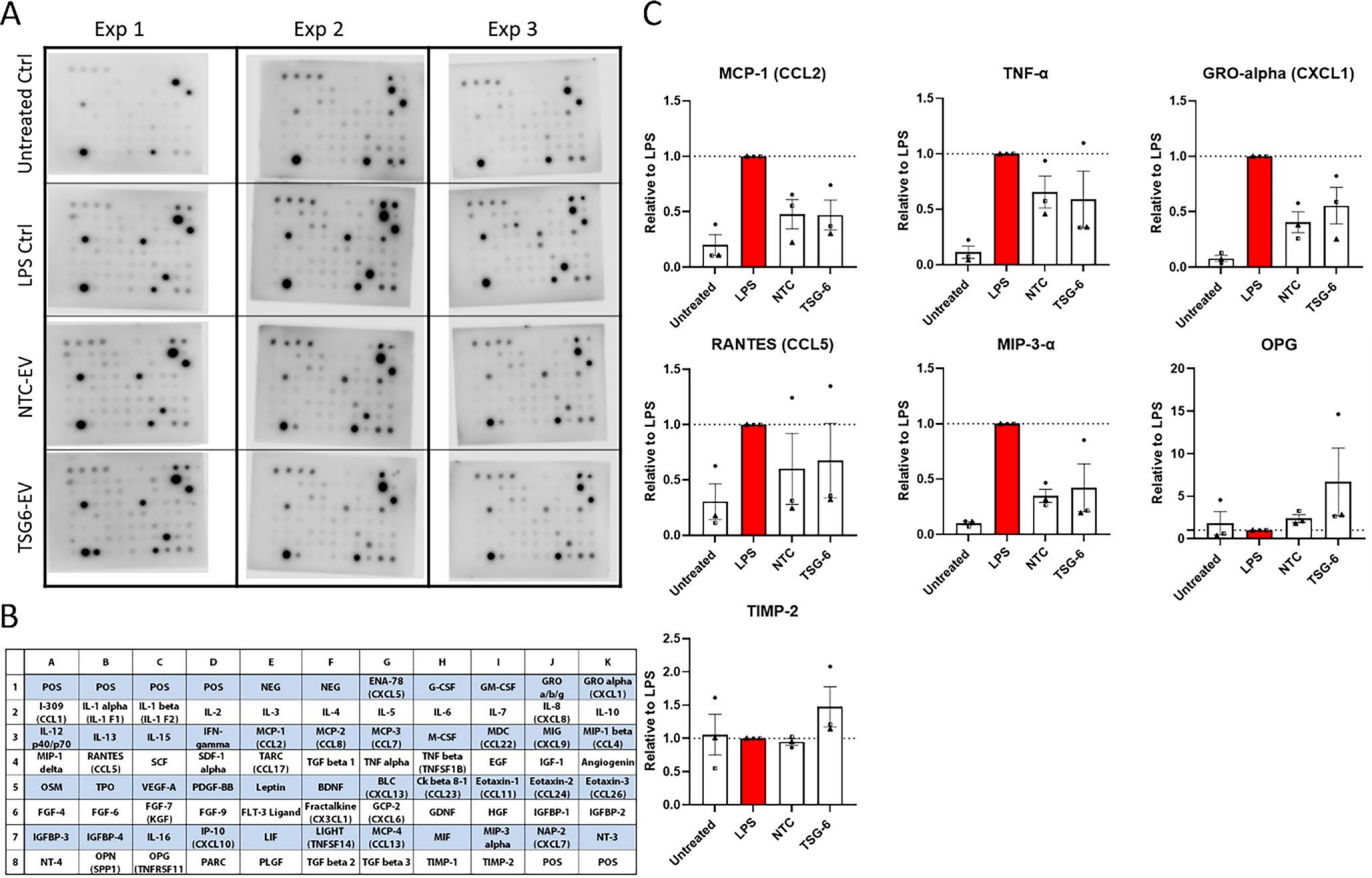
The effects of LPS + MSC-EV cotreatments on macrophage cytokine secretion. (**A**) Cytokine arrays of macrophages. (**B**) Reference table of each cytokine blot location (Adapted from manufacturer https://www.raybiotech.com/human-cytokine-array-c5-aah-cyt-5). (**C**) Semiquantitative changes in cytokine secretion relative to LPS treatment control. (Experimental conditions include: Untreated = no LPS, no EVs; LPS = plus LPS, no EVs; NTC = plus LPS, plus NTC-EVs at 1000 particles/cell; TSG-6 = plus LPS, plus TSG-6-EVs at 1000 particles/cell) (n = 3), mean ± SEM

To determine that the previously observed changes in on the mRNA level are also observed on the protein level, cytokine secretion was assessed with a Cytokine Antibody Array C5. We observed a significant downregulation of secreted CCL2, MIP-3-alpha, and CXCL1 upon MSC-EVs treatment, with comparable effects induced by TSG-6 activated EVs and NTC-EVs. The cytokines TNF-α and CCL5 presented partial downregulation (Fig. 9). This is consistent with the observed reduction of the pro-inflammatory gene expression. On an interesting note, TSG-6 EVs co-treatments induced a visible (but non-significant) increase in cytokines OPG and TIMP-2, which are known to have tissue-protective effects [97].

## Discussion

Recent studies have suggested that MSC-EVs have great potential in the treatment of multiple pathologies [14, 15, 36, 59, 71, 83, 98, 99]. We have previously used a CRISPR-activation approach to develop a line of human MSCs overexpressing gene TSG-6 [78]. The EVs secreted by these MSCs presented altered protein cargo relative to control MSC-EVs while retaining typical MSC-EV characteristics. Furthermore, these EVs attenuated the pro-inflammatory gene expression in an in vitro intervertebral disc cell model.

Considering the large impact macrophages have in a wide range of human inflammatory pathologies, we now aimed to determine the impact of MSC-EVs in an in vitro LPS-stimulated macrophage model, by observing EV uptake into macrophages, and assessing changes to gene expression and cytokine secretion. We especially aimed to compare the effect of CRISPR-engineered EVs from the TSG-6 “activated” MSCs to NTC-MSC EVs. Additionally, we performed multiple EV dose comparisons to assess whether macrophages respond to EV treatment in a dose-dependent manner. Lastly, and complimentary to our previous proteomic analysis of EV cargo, we further analyzed the miR cargo in EVs through small RNA-sequencing to comprehensively assess changes in EV cargo material after CRISPR activation.

For the current study, we harvested EVs from the same cell line we used in the past, TSG-6 activated ASC52telo human MSCs (using appropriate negative controls), and validated the long-term TSG-6 overexpression. To obtain EV yields necessary for dose-dependency experiments, we opted to harvest EVs using the Total Exosome Isolation Reagent. We were able to verify that harvested EVs maintained characteristics similar to those we obtained from ultracentrifugation, and consistent with those characteristics widely reported in the literature. We furthermore validated the successful uptake of PKH26 dyed EVs by macrophages stimulated with LPS.

The immune-modulatory capacity of our EVs was tested in LPS-stimulated THP-1 derived macrophages, which is a common in vitro model and it has been used by previously by others to assess the effect of EV treatments [57–59]. Our main finding is that the treatment of macrophages with TSG-6 activated MSC-EVs exerts a significant downregulation of multiple pro-inflammatory markers. More specifically, when testing one standard EV dosage for all samples, we observed statistically significant downregulations in the gene expression of IL-1β, CCL2, CXCL10, and TNF-α. Interestingly, we observed a trend toward stronger downregulation of genes IL-1β and TNF-α when using TSG-6 activated EVs compared to NTC-EVs, suggesting a potential enhancement in anti-inflammatory effects. When testing different EV dosages, we confirmed the down-regulation of IL-1β, IL-6, CCL2, and TNF-α, with a partial dose-response effect. Higher dosages tended to cause larger downregulation for CCL2 and TNF-α, albeit this effect did not reach statistical significance. Consistent with these overall gene expression results, cytokine secretion was also downregulated for several pro-inflammatory cytokines, including CCL2. CCL2 plays a key role in the migration and infiltration of monocytes and macrophages, and has further roles in the immune response, and several pathologies [100]. In this regard, MSC-EVs are known to inhibit CCL2, and modulate macrophage inflammatory activity, and polarize to M2 phenotype by binding CCL2 through receptor CCR2 present in MSC-EVs [101, 102].

Similar to our previous proteomic analysis that showed EV protein cargo differences upon CRISPR modification, we now also observed altered expression levels of multiple miR in EVs from TSG-6 activated MSCs, relative to NTC and WT controls. Of note, significant changes were also reported in the “NTC vs WT” comparisons, which is consistent with our proteomic findings. For “TSG-6 vs NTC” comparisons, 15 differentially expressed miRs were identified by small-RNA sequencing, whereby 6 of these miRs were overexpressed in TSG-6 activated EVs. Further analysis of these miRs highlighted “miR-181a-5p” as a potential target of gene TSG-6. The relevance of miR-181 in stem cell biology and immunomodulation has previously been highlighted. For example, exposure of adipose-derived stem cells to TNF-α not only causes upregulation of TSG-6 expression but also of miR-181a [103]. Furthermore, miR-181a-5p and miR-181b-5p play an important regulatory role in various inflammatory processes, including vascular inflammation, atherogenesis, arterial hypertension, and colitis [104–107]. MSC-EVs containing miR-181a-5p were shown to inhibit microglial inflammation [108], and overexpression of miR-181b-5p was able to downregulate IL-6 in cementoblasts of a mouse model [109].

Our own sequenced-based target predictions showed that miR-181a-5p targets multiple genes in pathways relevant to macrophage stimulation, including TGF-beta signaling, TNF-α signaling, NF-kappa B signaling, FoxO signaling, T cell receptor signaling, inflammatory mediator regulation of TRP channels, apoptosis, and cellular senescence. Another over-expressed miR, “miR-181b-5p” presented similar results. These pathways play key roles in the regulation of inflammatory response and other pathophysiological processes, by promoting macrophage polarity towards M1 or M2 phenotype, modulating pro- or anti- inflammatory activity, macrophage migration, and survival [25, 28, 110–115].

The integration of miRNA-target prediction with protein-protein interaction data provides new insight into how TSG-6 activation shapes MSC-derived EV cargo and downstream signaling. Within our network, TSG-6 (TNFAIP6) is directly connected to both aggrecan (ACAN) and RANKL (TNFSF11), which are in turn linked to miR-181a-5p and miR-181b-5p, respectively - two miRNAs found to be upregulated in EVs from TSG-6-activated MSCs.

Our data suggest that TSG-6 activation promotes the selective enrichment of miR-181a-5p and miR-181b-5p into MSC-derived EVs, forming part of a regenerative and immunomodulatory cargo profile. This may be mediated through TSG-6’s interaction with extracellular matrix components like aggrecan, which could influence intracellular signaling pathways and enhance packaging of miR-181a-5p into EVs [116, 117]. In parallel, TSG-6’s inhibitory effect on RANKL (TNFSF11) may suppress downstream pro-inflammatory signaling such as NF-κB in MSCs, thereby shifting intracellular pathways that regulate EV content [118–120]. This altered signaling environment could favor the transcription or selective packaging of miR-181b-5p, aligning with a broader anti-inflammatory and immunoregulatory EV profile. Together, these axes suggest that TSG-6 orchestrates specific EV miRNA signatures by integrating extracellular cues with immune-regulatory signaling, potentially priming MSC-EVs to influence both matrix remodeling and macrophage responses.

Supporting this axis, we observed a trend toward increased osteoprotegerin (OPG) release in macrophages treated with EVs from TSG-6-activated MSCs, compared to NT EVs. As OPG acts as a decoy receptor for RANKL (TNFSF11), its upregulation may reflect a shift toward a regulatory macrophage phenotype. Given RANKL’s emerging role in promoting inflammatory signaling and immune cell activation beyond bone (ref), this finding suggests that TSG-6-EVs may modulate not only cytokine responses but also RANKL-mediated inflammatory crosstalk, contributing to a broader anti-inflammatory program [121, 122]. While RANKL-OPG signaling is best known in bone homeostasis, its involvement in macrophage activation and chronic inflammation is increasingly recognized, aligning with the observed immunomodulatory effects of TSG-6-EVs [121, 122].

KEGG and GO analysis shows the enrichment of key signaling and immune-related pathways related to upregulated miRNAs in macrophages. Activation of the MAPK, Jak-STAT, and T cell receptor signaling pathways points to enhanced cytokine production and immune cell communication, while enrichment of p53 signaling and stress-activated kinase cascades suggests a cellular stress response. The observed changes in transcriptional regulation and metabolic processes, further support functional reprogramming of macrophage inflammatory states. Enrichment of pathways related to hypoxia and viral response highlights macrophages’ adaptability within diverse microenvironments, such as infected or tumor-associated tissues. In summary, these findings suggest that the differentially expressed genes drive a coordinated transcriptional program promoting immune activation, cellular adaptation, and environmental sensing.

Overall, our network-based analysis, combined with miRNA cargo profiling and macrophage functional assays, supports a model in which TSG-6 activation leads to coordinated upregulation of miR-181a-5p and miR-181b-5p in EVs, with potential relevance to ECM homeostasis and immune regulation. These effects may be mediated by interactions between TSG-6, aggrecan, RANKL, and OPG, forming a multilayered mechanism of inflammation resolution and tissue remodeling.

TSG-6 is produced by different cell types (including MSCs, fibroblasts, and immune cells) in response to pro-inflammatory cytokines such as TNF-α, with multiple functions and a wide range of ligands [81]. In the case of MSCs, TSG-6 has an autocrine effect, and loss of TSG-6 expression leads to changes in cell morphology, proliferation, loss of multilineage differentiation potential, changes in cytokine release profiles, and changes to transcription factor expression [80]. Although our proteomic and small RNA sequencing data supports the hypothesis that TSG-6 overexpression leads to downstream changes in MSC-EV contents, it is still unclear to what extent this occurs, and the exact mechanisms involved. To further investigate the effects of TSG-6 on MSC-EV proteome of miR contents, a complete functional knockout of TSG-6 in the same MSC line (ASC52telo) could be used, using CRISPR-Cas9.

Considering the potential shown by CRISPR-activation of MSCs, future works could explore additional targets. For example, Kim et al. (2020) boosted the immunosuppressive effects of MSC-EVs by transfecting MSCs with plasmids for TGF-b1, PTX3, let-7b-5p, and miR-21-5p [123]. Other experiments reported various therapeutic effects after overexpressing other miRs, such as miR-let7c, miR-124, or miR-146b, among others [124–126]. In addition to augmenting the therapeutic effect of MSC-EVs, increasing the EV secretion capacity of MSCs is of high relevance to enhance their translational potential. For example, Kojima et al. (2018) reported increasing the production of EVs in HEK293T cells by overexpressing STEAP3, SDC4, and NadB [127], and a similar approach could be used for MSCs using CRISPR activation. With CRISPR SAM activation, accurate and robust overexpression of endogenous genes can be achieved, and additionally, CRISPR-activation can be further used for simultaneous multiplexed activation of multiple genes of interest, which could be a very powerful tool for boosting MSC-EV based therapeutics [77]. In our current study, we used THP-1 derived macrophages, which are an excellent model system, but do not perfectly mimic the properties of in vivo macrophages on human pathology. To better study the relevant pathophysiological effects, future studies will seek to use primary macrophages isolated from healthy donors, and diseased patients. Other future studies could include time-resolved cytokine profiling to assess the kinetics of macrophage polarization in response to MSC-EV treatment. While our current data demonstrate a reduction in pro-inflammatory markers at a single time point, macrophage polarization toward an M2-like phenotype is a dynamic process involving sequential cytokine changes. Temporal profiling would help clarify the duration and stability of the EV-induced immunomodulatory effects, differentiate between immediate and downstream responses, and reveal whether TSG-6-activated EVs accelerate or enhance this transition compared to controls. Additionally, a mouse model of LPS-induced inflammation could be used to test the effect of MSC-EVs in vivo.

In summary, our work demonstrates that MSC-EVs have a significant effect on the gene expression and cytokine secretion of LPS-induced macrophages. Additionally, TSG-6 activated MSC-EVs show a trend of superior effectiveness relative to control, although it does not reach statistical significance. Furthermore, CRISPR-activation of TSG-6 can alter both EV protein and EV miR contents, which opens up many potential options for nanodelivery of EV-contained therapeutic biomolecules.

## Supplementary Information

Below is the link to the electronic supplementary material.

Supplementary Material 1 (XLSX 301 KB)

Supplementary Material 2 (DOCX 405 KB)

## Data Availability

The data supporting this article have been included as part of the manuscript or supplementary information files. Additional data for this article, including the miR sequencing data have been deposited to the Gene Expression Omnibus repository with the GEO accession GSE294611. All other datasets are available in the Figshare repository (https://doi.org/10.6084/m9.figshare.28765652.v1).
