# Supplementary material for: TSG-6 Activated MSC-derived Extracellular Vesicles Present Altered micro-RNA Contents and Ameliorate the Inflammatory Phenotype of Macrophages in Vitro
Source: Inflammation. 2026 Jan 13;49(1):42. doi: 10.1007/s10753-025-02398-y (PMC12862027; doi:10.1007/s10753-025-02398-y)
Supplement: Supplementary file 2 — Supplementary Material 2 (DOCX 405 KB) [file 10753_2025_2398_MOESM2_ESM.docx]

Supplementary Information for:

TSG-6 activated MSC-derived Extracellular Vesicles present altered micro-RNA contents and ameliorate the inflammatory phenotype of macrophages in vitro

Iker Martinez-Zalbidea,^a^ Alyssa Rzasa,^a^ Varun Puvanesarajah,^b^ Wolfgang Hitzl,^c,d,e^ and Karin Wuertz‑Kozak,^a,f^

1. Department of Biomedical Engineering, Rochester Institute of Technology (RIT), Rochester, NY, USA
2. Department of Orthopedics and Rehabilitation, University of Rochester Medical Center, Rochester, NY, USA
3. Research and Innovation Management (RIM), Paracelsus Medical University, Salzburg, Austria
4. Department of Ophthalmology and Optometry, Paracelsus Medical University, Salzburg, Austria
5. Research Program Experimental Ophthalmology and Glaucoma Research, Paracelsus Medical University, Salzburg, Austria
6. Schön Clinic Munich Harlaching, Spine Center, Academic Teaching Hospital and Spine Research Institute of the Paracelsus Medical University Salzburg (Austria), Munich, Germany

Corresponding author:

Prof. Karin Wuertz-Kozak (kwbme@rit.edu)


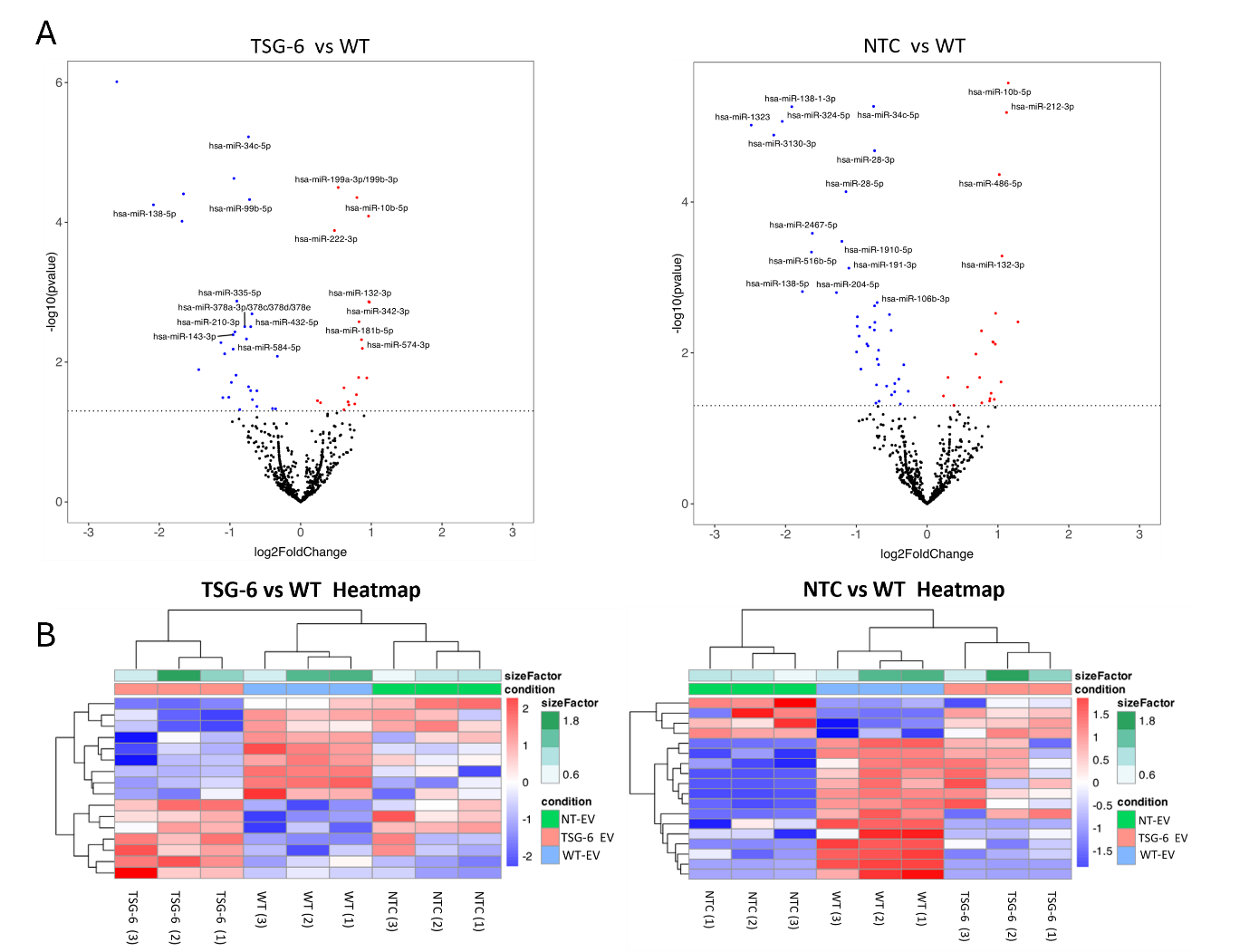


**Supplementary Fig. S1** (A) Volcano-plot comparison of EV miRs in TSG-6 vs WT” and “NTC vs WT” groups. The plots use a significance threshold of p < 0.05 (–log₁₀(p) > 1.301) on the y-axis and log₂ fold change thresholds of >1 and <–1 on the x-axis. (B) Heat map of sample size factors including all EV samples.
